# Ultra-small and highly dispersive iron oxide hydroxide as an efficient catalyst for oxidation reactions: a Swiss-army-knife catalyst

**DOI:** 10.1038/s41598-021-85672-x

**Published:** 2021-03-23

**Authors:** Mojtaba Amini, Younes Mousazade, Zahra Zand, Mojtaba Bagherzadeh, Mohammad Mahdi Najafpour

**Affiliations:** 1 Department of Chemistry, Faculty of Science, University of Maragheh, Golshahr, P.O. Box. 55181-83111731, Maragheh, Iran; 2 Department of Inorganic Chemistry, Faculty of Chemistry, University of Tabriz, Tabriz, Iran; 3 Department of Chemistry, Institute for Advanced Studies in Basic Sciences (IASBS), Zanjan, 45137-66731 Iran; 4 Chemistry Department, Sharif University of Technology, P.O. Box 11155-3615, Tehran, Iran; 5 Center of Climate Change and Global Warming, Institute for Advanced Studies in Basic Sciences (IASBS), Zanjan, 45137-66731 Iran; 6 Research Center for Basic Sciences and Modern Technologies (RBST), Institute for Advanced Studies in Basic Sciences (IASBS), Zanjan, 45137-66731 Iran

**Keywords:** Heterogeneous catalysis, Inorganic chemistry

## Abstract

Ultra-small and highly dispersive (< 10 nm) iron oxide hydroxide is characterized by some methods. The compound is an efficient and stable catalyst for alcohol oxidation, organic sulfide oxidation, and epoxidation of alkenes in the presence of H_2_O_2_. The electrochemical oxygen-evolution reaction of the iron oxide hydroxide is also tested under acidic, neutral, and alkaline conditions. In the presence of the iron oxide hydroxide, excellent conversions (75–100%) and selectivities of substrates (92–97%), depending on the nature of the sulfide, were obtained. Benzylalcohols having electron-donating and-withdrawing substituents in the aromatic ring were oxidized to produce the corresponding aldehydes with excellent conversion (65–89%) and selectivity (96–100%) using this iron oxide hydroxide. The conversion of styrene and cyclooctene toward the epoxidation in the presence of this catalyst are 60 and 53%, respectively. Water oxidation for the catalysts was investigated at pH 2, 6.7, 12, and 14. The onset of OER at pH 14 is observed with a 475 mV overpotential. At 585 mV overpotential, a current density of more than 0.18 mA/cm^2^ and a turnover frequency of 1.5/h is observed. Operando high-resolution visible spectroscopy at pH 14, similar to previously reported investigations, shows that Fe(IV)=O is an intermediate for water oxidation.

## Introduction

Iron oxides are compounds suited for different applications^1–5^. The use of iron oxides as natural pigments has taken place since prehistoric times^1^. Iron oxides are excellent adsorbents for lead removal from aquatic media^1^. The compounds are reported as catalysts for many reactions, specifically, magnetite, and hematite are among important catalysts for the oxidation/reduction and acid/base reactions^1–5^. Recently, iron oxides in a pure form or mixed with other metal oxides have been reported as catalysts for CO oxidation, oxygen-evolution reaction (OER), medical diagnostics, organic compounds oxidation, or degradation reactions^1–6^. Nanoparticle iron oxides are even more effective than micron-sized iron oxides^1–6^. There are many procedures for the oxidation of organic compounds to synthesize important molecules using Fe compounds. Fe(acac)_2_ (acac: acetylacetonate) was used for selective oxidation of sulfide to sulfoxide in the presence of molecular oxygen^7^. Iron(II) acetylacetonate/SiO_2_/Fe_3_O_4_ was reported to be a recoverable heterogeneous nanocatalyst for selective oxidation of sulfides to sulfoxides using 30% hydrogen peroxide^8^. Fe(III) oxide nanoparticles supported on mesoporous silica was also reported for the chemoselective oxidation of sulfides to sulfoxides by hydrogen peroxide^9^. Wang's group reported that Fe(II) exchanged NaY zeolite are efficient catalysts for the epoxidation of alkenes with molecular oxygen as an oxidant^10^. α-Fe_2_O_3_ nanoparticles effectively catalyzed the epoxidation of alkenes using molecular oxygen^11^.

Beller's group reported that unsupported nano-γ-Fe_2_O_3_ was an excellent, stable, and highly selective catalyst for the oxidation of olefins and alcohols to aldehydes using hydrogen peroxide as an oxidant^12^. Alcohol oxidation by iron oxides was reported by some research groups^13–15^.

Iron-based films have been studied as electrocatalysts for OER^6^. However, the preparation of such films by electrodeposition is not easy since Fe(III) ions will easily precipitate under neutral conditions^16^. Lyons and Doyle reported OER by Fe oxides. They showed that OER depended on the conditions under which the iron oxide film was generated^16^.

An ultrathin Fe oxide-based film was reported, which showed a turnover number of 5.2 × 10^4^. The low Fe loading (12.3 nmol cm^−2^ on indium tin oxide electrode) was necessary for the efficiency of this catalyst^17^.

The reduction of Fe(VI) to Fe(III) for synthesizing a unique Fe oxide on the surface of fluorine-doped tin oxide (FTO) electrode was reported^18^. The electrode could be used as stable water-oxidizing anodes at pH = 13 to yield current densities of 1 mA cm^−2^ at an overpotential of 550 mV^18^.

Herein, we used the commercial ultra-small (< 10 nm) iron oxide hydroxide (FeOOH) as an efficient catalyst for alcohol oxidation, organic sulfide oxidation, and epoxidation of alkenes in the presence of H_2_O_2_. The catalyst was evaluated for OER under acidic, neutral, and alkaline conditions. The commercial FeOOH is promising to be used as catalysts for different reactions because there are easily accessible at competitive price and high quality.

## Results

The highly dispersive iron oxide hydroxide (**1**) is pure (99.5% trace metals basis) and highly dispersed (20 wt% in water).

**Figure 1:**
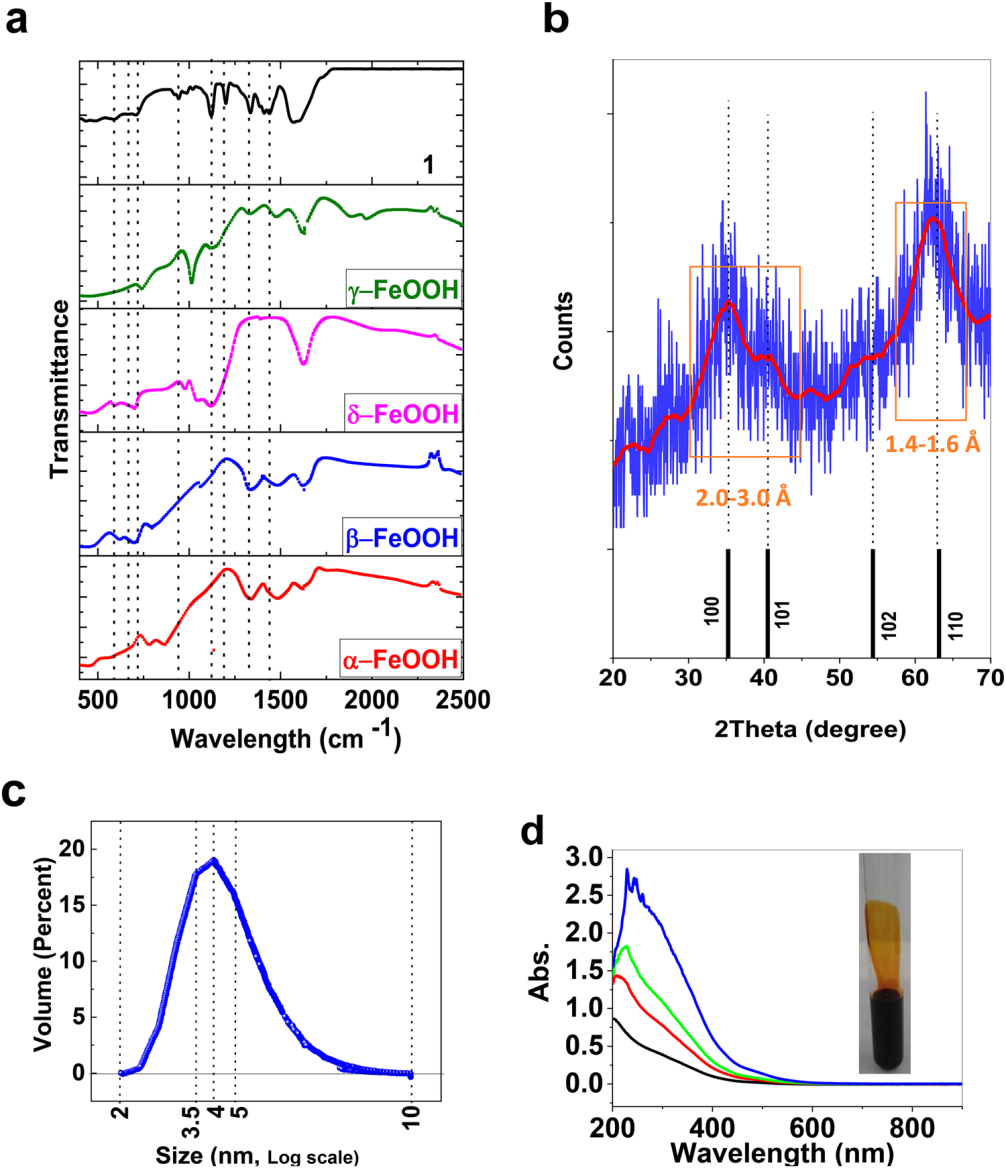
FTIR spectra of α-FeOOH, β-FeOOH, γ-FeOOH, δ-FeOOH, and **1**. The data of FTIR spectra for different FeOOH phases are from ref. 20 (**a**). XRD patterns for **1** (blue) and δ-FeOOH (black) (Ref. code.: 00–013-0518) (**b**). The smoothing XRD pattern for **1** is shown in red (**b**). DLS for **1** (13 μM) (**c**). UV–Visible spectra of **1** 22.4 nM (black), 33.6 nM (red), 44.8 nM (green), and 67.2 nM (blue) (**d**). Insert showed a photograph of **1** in a test tube.

At FTIR spectra of **1**, a broad band at ~ 3300–3500 cm^−1^ related to antisymmetric and symmetric O–H stretchings, and at ~ 1620–1630 cm^−1^ related to H–O–H bending were observed (Fig. 1a; Fig. S1)^19,20^. Mei et al. studied FTIR spectra of α-, β-, γ- and δ-FeOOH and outlined the features attributed to FeO_6_ groups in these compounds at 400–1100 cm^−1^ (Fig. 1a). It was reported that the FTIR bands at 883 and 795 cm^−1^ were related to the –OH bending modes in α-FeOOH (Fig. 1a)^20,21^. The bands at 847 and 696 cm^−1^ were attributed to the –OH bending modes in β-FeOOH (Fig. 1a)^22^. The bands at 1020 and 750 cm^−1^ were the bending vibration of –OH modes in γ-FeOOH (Fig. 1a)^23^ and finally, the bands at 575, 650, 710 and 1120 cm^−1^ were the bending vibration of OH modes in δ-FeOOH (Fig. 1a)^24^. Among these iron oxide hydroxides, the FTIR spectrum of **1** was similar to δ-FeOOH, and its attributed peaks were observed at 575, 650, 710, and 1120 cm^−1^ (Fig. 1a). The peak at 1200–1600 cm^−1^ could be related to surfactants.

X-ray powder diffraction (XRD) is a fast analytical method for phase identification of a crystalline material and information on unit cell dimensions. The analyzed material should be finely ground, homogenized, and the average bulk composition is determined. X-ray diffraction is a common technique for the study of crystal structures and atomic spacing.

X-ray diffraction works based on constructive interference of monochromatic X-rays and a crystalline sample. X-ray is generated by a cathode ray tube, filtered to the monochromatic radiation, collimated to concentrate, and focused on the sample. The interaction of the ray with the sample produces constructive interference, Bragg's Law, where (Eq. 1):

1 $${\text{n}}\uplambda = 2{\text{d}}\sin\uptheta$$

This law relates the wavelength of electromagnetic radiation to the diffraction angle and the lattice spacing in a crystalline sample. These diffracted X-rays are then detected, processed, and counted. By scanning the sample through a range of 2θ angles, all possible diffraction directions of the lattice should be attained due to the random orientation of the powdered material. According to the conversion of the diffraction peaks to d-spacings, the identification of the crystalline material is possible because each crystalline material has a set of unique d-spacings.

XRD showed that **1** was not crystalline, but the attributed weak peaks for δ-FeOOH (Fig. 1b) (Ref. code.: 00-013-0518; crystal system: Hexagonal; a (Å): 2.9410; b (Å): 2.9410; c (Å): 4.4900; α (°): 90.0000; β (°):90.0000; γ (°): 120.0000; volume of cell: 33.63 Å^3^) were observed for **1**. The size of **1** was calculated at ca. 4–7 nm using the Scherrer equation^25^. The size of the nanoparticle was 2–8 nm based on DLS (Fig. 1c). **1** was also highly monodisperse in size. We found that **1** was stable at least for three years when stored at the pH range (3.0–4.0) and 2–8 °C without any aggregation or agglomeration (Fig. 1c).

UV–Vis spectrum of** 1** showed a broad peak at 300–400 nm, which was due to ligand to metal charge transfer (Fig. 1d). However, Sherman et al. suggested that the ligand to metal charge transfer transitions in Fe(III) oxides and silicates occur at higher energies than those suggested by others and that the visible region is an intense Fe(III) ligand field as well as Fe(III)-Fe(III) pair transitions^26^. They suggested that both types of these transitions are Laporte and spin-allowed via the magnetic coupling of adjacent Fe(III) cations^26^.

**Figure 2:**
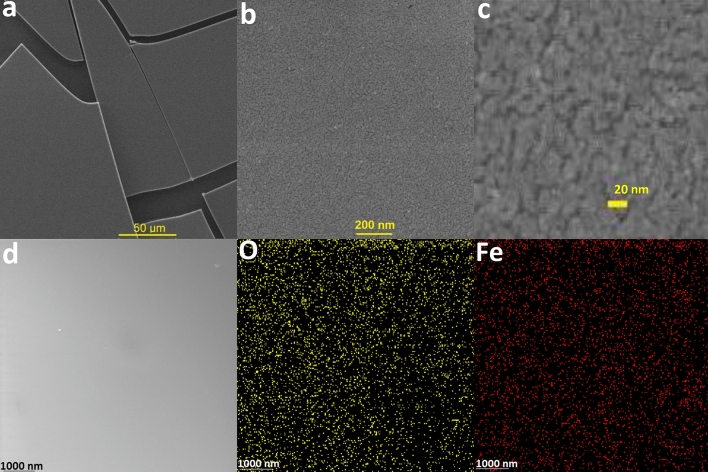
SEM images of **1** at different magnifications (**a**–**c**). EDX-Mapping for oxygen (yellow) and iron (red) on the surface of **1** (**d**).

A scanning electron microscopy (SEM) is a type of electron microscopy that provides images of a sample by scanning the surface with the electron beam. The interaction of electrons with the surface atoms in the sample forms signals with information on the surface topography and composition of the sample. Samples are investigated in high vacuum in a conventional SEM or wet conditions or environmental SEM. SEM images of **1** showed small nanoparticles (Fig. 2a and b). A high monodispersity of particles was observed in SEM images (Fig. 2a and b). However, the resolution of a SEM is about 10 nm (nm), which is limited by the width of the electron beam and the interaction volume of electrons in a sample. Thus, tiny particles in Fe oxide are not clearly detectable by SEM (Fig. 2c). EXD-Mapping and EDX spectrum showed high dispersity for Fe and O on the surface of **1** (Fig. 2d; Fig. S2).

**Figure 3:**
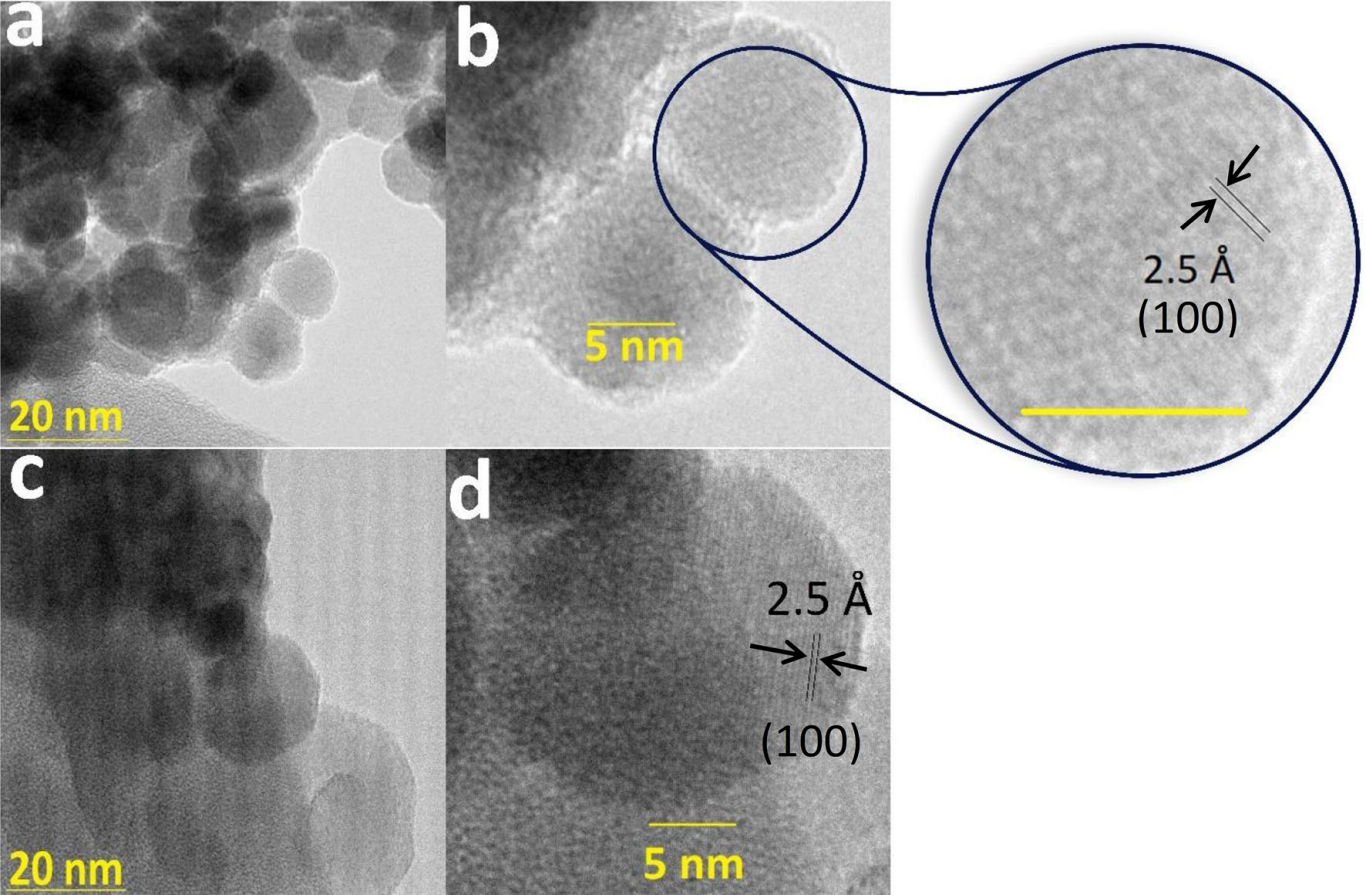
TEM images for **1** at different magnifications before (**a**, **b**) and after the methylphenyl sulfide oxidation (**c**, **d**). Scale bar for inset is 5.0 nm.

Transmission electron microscopy (TEM) is a microscopy method in which a beam of electrons is transmitted through a sample to form an image. The sample should have an ultrathin section less than 100 nm thick or a suspension on a grid. In TEM, an image is formed from the interaction of the electrons with the sample as the beam is transmitted through the specimen. The resolution of a TEM is 25–50 times greater than SEM. In TEM images for **1**, amorphous and tiny nanoparticles (< 10 nm) and high monodispersity were observed (Fig. 3a; Fig. S3). HRTEM images showed a crystal lattice spacing of 2.5 Å, corresponding to (100) plan of δ-FeOOH (Fig. 3b). After the methylphenyl sulfide oxidation (next section), the catalyst showed no change in the morphology, phase, or size (Fig. 3c and d), which show the catalyst is stable.

## Discussion

### Catalytic performance of 1

The study of catalytic performance began with an effort to optimize the reaction conditions for sulfide oxidation. Methylphenylsulfide as a model substrate, and H_2_O_2_ as a green oxidant were used to optimize the sulfoxide production (Table 1). Water, as a standard “green” solvent, was selected for all oxidation reactions. In the absence of a catalyst as a blank experiment (entry 1), a trace amount of products was observed, which indicated that the presence of a catalyst is crucial. By continuously increasing the catalyst amount from 22.5 to 67.4 μL (entries 2 − 4), a significant increase in the conversion was observed. As indicated in Table 1 (entries 5–7), a substantial decrease in conversion was observed by reducing the amount of oxidant.

**Table 1:** The effect of various conditions on the methylphenyl sulfide to sulfoxide for 4 h in the presence of H_2_O_2_ as an oxidant and water as solvent^a^.

Entry	Amount of catalyst (μM)	Oxidant amount (mmol)	Conversion (%)	Selectivity (%)
1	–	0.4	11	100
2	22.5	0.4	49	99
3	44.9	0.4	81	96
4	67.4	0.4	100	92
5	67.4	0.3	84	97
6	67.4	0.2	58	98
7	67.4	0.1	37	100

^a^GC yield.

To examine the substrate scope of the reaction, the optimized reaction conditions were then extended to a range of different sulfides, including dialkylsulfides, cyclic sulfide, benzylalkylsulfide, and dialkylsulfides, with H_2_O_2_ as a green oxidant (Table 2). After the methylphenyl sulfide oxidation, the catalyst showed no change in the morphology, phase, or size (Fig. 3).

**Table 2:** Oxidation reactions catalyzed by **1**^a^.

Entry	Oxidation reaction	Conversion (%)^b^	Selectivity (%)^c^	TON (TOF(h^−1^))
1		100	92	2967 (741)
2		91	94	2700 (675)
3		99	95	2938 (734)
4		100	93	2967 (741)
5		100	95	2967 (741)
6		75	97	2226 (556)
7		89	96	2641 (660)
8		71	99	2107 (527)
9		85	98	2522 (631)
10		65	99	1929 (482)
11		80	100	2374 (593)
12		58	100	1721 (430)
13		60	78	1780 (445)
14		53	100	1573 (393)

^a^Reaction conditions; four hours; catalyst (67.4 μM), H_2_O (1 mL), substrate (0.2 mmol), H_2_O_2_ (0.4 mmol), at room temperature.

^b^The GC conversion (%) is measured relative to the starting substrate.

^c^Selectivity to sulfoxide = (sulfoxide %/(sulfoxide% + sulfone%)) × 100; Selectivity to benzaldehyde = (aldehyde%/(aldehyde% + carboxylic acid%)) × 100; Selectivity to epoxide = (epoxide%/(epoxide% + aldehyde%)) × 100; TON = mol product/mol catalyst; TOF = TON/time of reaction (4 h).

Similar to the oxidation of methylphenylsulfide, excellent catalytic activities, and selectivities were obtained for all the sulfides tested (entries 1–6). Excellent conversions (75–100%) and selectivities of substrates (92–97%), depending on the nature of the sulfide, were obtained for all cases. The diversity of oxidation reaction catalyzed by **1** was also extended to oxidation of various alcohols. Benzylalcohols having electron-donating and -withdrawing substituents in the aromatic ring were oxidized to produce the corresponding aldehydes with excellent conversion (65–89%) and selectivity (96–100%) (entries 7–10). Furthermore, steric hindrance had little effect on the reaction yields because of the ortho substituents in the benzylalcohols (entries 8 and 9). Secondary alcohols such as 1-indanol and cyclohexanol could be converted to the corresponding ketones in 80% and 58% conversion, respectively (entries 11 and 12). These results encouraged us to check the epoxidation reaction of several alkenes in the presence of **1**, but the catalytic epoxidation of styrene and cyclooctene were found to be less efficient than that of sulfide/alcohol oxidation (entries 13 and 14).

According to the nature of the oxidation products, the mechanism of reactions was proposed. In the presence of H_2_O_2_, after the formation of Fe(IV)=O center, the reaction of organic substrates to a Fe(IV)=O could be proposed as the mechanism for the oxidation reactions in the presence of **1** (Fig. S4). To show the advantage and performance of the present catalytic system in comparison with lately reported protocols, we compared the results of the benzyl alcohol oxidation in the presence of other nano-iron oxide catalysts^12^. As shown in Table 3, in contrast to previously reported systems, the catalytic system presented in this paper does not suffer from the severe reaction conditions, such as using a large amount of catalyst, long reaction time, and high reaction temperature.

**Table 3:** Oxidation of organic substrates with different nano-iron oxide catalysts.

Entry	Catalyst	Condition	Conversion (%)	Selectivity (%)	References
1	Ultra-small FeOOH	67.4 μmol catalyst, 0.4 mmol H_2_O_2_, 0.2 mmol substrate, room temperature, 4 h	89	96	This work
2	Bulk α-Fe_2_O_3_	1 mol% of catalyst, 10 mmol of substrate, 10 mmol H_2_O_2_, 75 °C, 12 h	5	99	^12^
3	Bulk γ-Fe_2_O_3_	1 mol% of catalyst, 10 mmol of substrate, 10 mmol H_2_O_2_,75 °C, 12 h	5	99	^12^
4	Nano γ-Fe_2_O_3_	1 mol% of catalyst, 10 mmol of substrate, 15 mmol H_2_O_2_, 75 °C, 12 h	72	66	^12^

### Oxygen-evolution reaction (OER)

**Figure 4:**
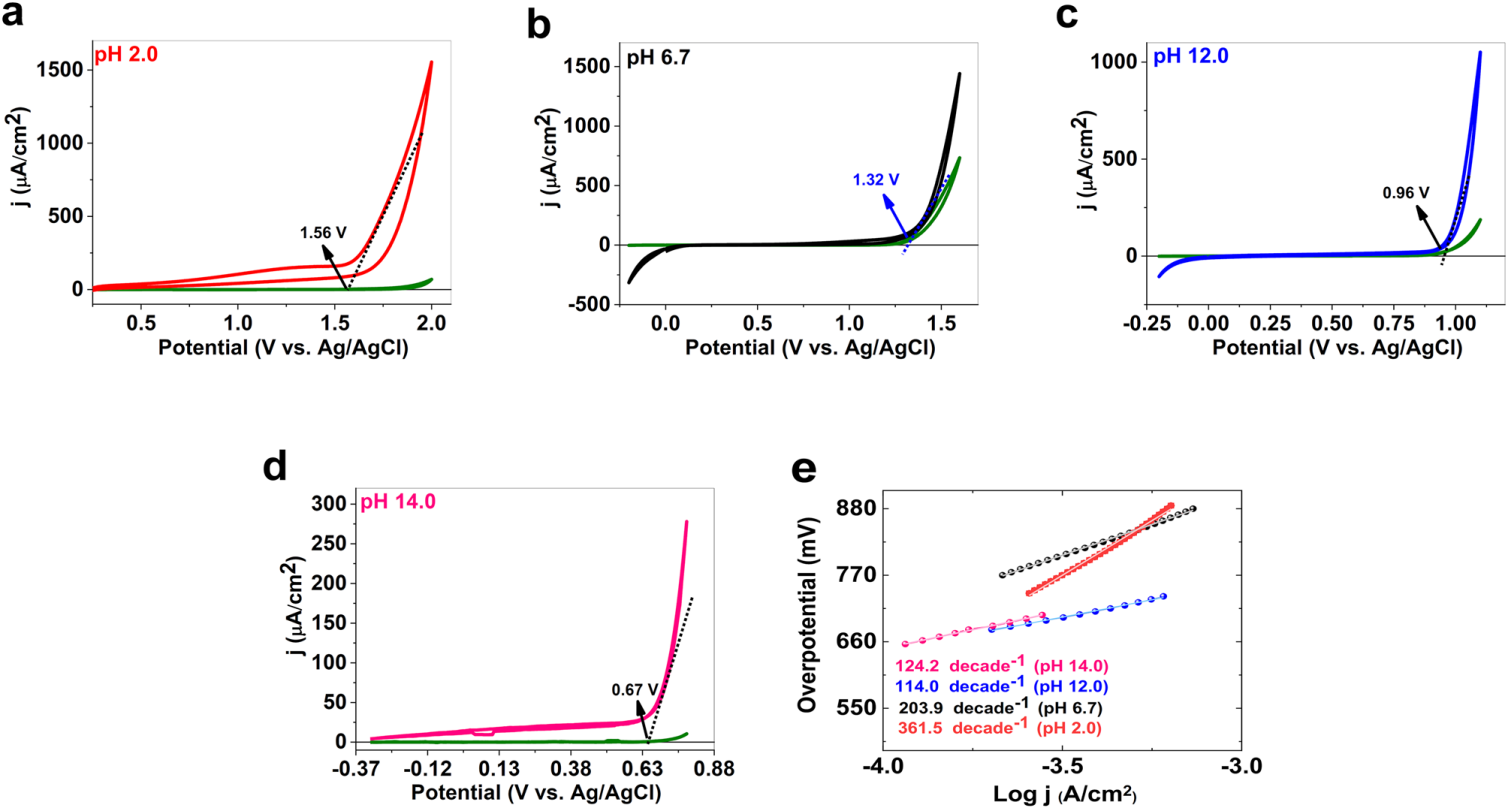
CVs (scan rate 25 mV/s) of **1** in pH 2 (phosphate buffer) (**a**), 7 (phosphate buffer) (**b**), 12 (phosphate buffer) (**c**) and 14 (KOH). Data for a bare FTO are shown in green. Tafel plots for **1** in pH 2 (red), 7 (black), 12 (blue), in bufferic phosphate solution and 14 (pink) in KOH.

OER of the catalyst in the stable potential ranges was investigated for the catalyst at pH 2, 6.7, 12, and 14 (Fig. 4). The onset of OER in the presence of a trace amount of **1** (≈ 1 mg (11.2 μmol), see ESI for details) using fluorine-doped tin oxide coated glass electrode (FTO) at pH 2 was observed at 1.56 V (throughout the remaining sections, all potentials are reported vs. Ag/AgCl (KCl (3 M) reference electrode) with 660 mV overpotential (Fig. 4a). At 1100 mV overpotential, a current density of more than 1.5 mA/cm^2^ and a turnover frequency of 1.35/h was observed. FTO showed low activity toward OER under the same conditions. The onset of OER in the presence of **1** at pH 6.7 was observed at 1.32 V with a 690 mV overpotential. At 870 mV overpotential, a current density of more than 1.45 mA/cm^2^ and a turnover frequency of 1.3/h were observed (Fig. 4b). The onset of OER in the presence of **1** at pH 12 was observed at 0.96 V with a 640 mV overpotential. At 680 mV overpotential, a current density of more than 1.0 mA/cm^2^ and a turnover frequency of 0.9/h was observed (Fig. 4c). Finally, the onset of OER in the presence of **1** at pH 14 was observed at 0.67 V with a 470 mV overpotential. At 580 mV overpotential, a current density of more than 0.18 mA/cm^2^ and a turnover frequency of 1.5/h was observed (Fig. 4d).

To compare oxygen-evolution activity and to find the reaction mechanism of electrocatalysts, a Log(A/cm^2^)/overpotential or Tafel plot is generally considered. Using the Tafel method, the sensitivity of the current to the applied potential is plotted, which provides information about the rate-determining steps. The Log(A/cm^2^)/overpotential or Tafel plots were recorded for **1** in all the stated conditions (Fig. 4e). Tafel slopes are often influenced by electron and mass transports, gas bubbles, etc. The slopes of Tafel plots for **1** at pH 2.0, 6.7, 12.0, and 14.0 using FTO were 361.5, 203.9, 114.0 and 124.2 mV∙decade^−1^, respectively, which suggests the electron transfer to the electrode is the rate-determining step. At pH 14 because of the production of FeO_4_^2−^ at higher potential, a different range was selected (Fig. 4). Table S1 shows a comparison of different metal-oxide based catalysts toward OER.

In the next step, an *operando* high-resolution visible spectroscopy was applied for a very thin and transparent FeOOH covered FTO. For Mn, Co, Ni, Fe, and Cu oxides, the changes in the oxidation state of the redox-active metal can be detected by the changes in the absorption in UV/Vis area. Such electrochromic character has been reported for materials based on metal oxides and related binary oxyhydroxides deposited on transparent substrate electrodes (ITO or FTO glass), where a broadband absorption was recorded upon oxidation of redox-active metal centers^27–29^.

For FeOOH, the *operando* high-resolution visible spectroscopy showed no peak below 0.53 V, but at 0.53 V a small peak at 475 nm was recorded; at higher potentials, in addition to this peak, other peaks at 560 and 660 nm were also observed. In our setup, the counter electrode was separated from the working electrode by a small salt-bridge to inhibit reaction hydrogen or other reductants to high-valent intermediates in the *operando* high-resolution visible spectroscopy (for setup see Fig. S5).

**Figure 5:**
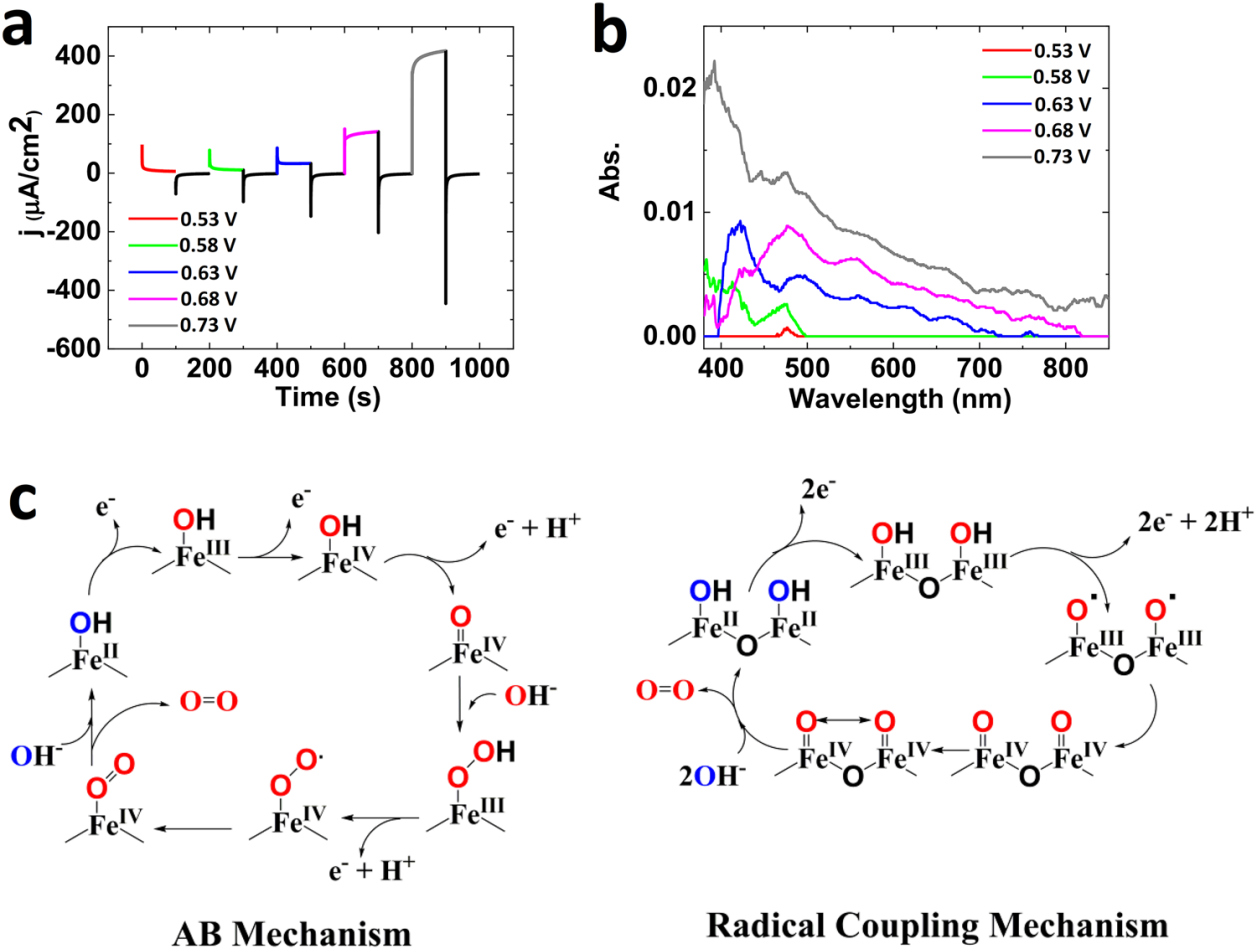
Operando high-resolution visible spectroscopy for Fe oxide covered FTO as working electrode (**a**, **b**) in KOH (pH 14). Each amperometry and its related high-resolution visible spectroscopy is in the same color (see Fig. S4 for setup). Two schematic proposed mechanisms for OER by Fe oxide under alkaline conditions (**c**).

The catalytic mechanisms for OER using Fe-based catalysts have been investigated by some research groups^30–33^. The reported mechanisms include acid–base and radical coupling mechanisms which in both the formation of Fe(IV)=O group is critical (Fig. 5c). A nucleophilic attack on Fe(IV)=O occurs by OH or H_2_O groups in acid–base mechanism^31^ while the radical coupling mechanisms include the reaction of two neighboring Fe(IV)=O groups^32^ and the O–O the bond formation is the rate-determining step (RDS). The Hamann’s group assigned the peak at 898 cm^−1^ to the Fe(IV)=O group on the surface of α-Fe_2_O_3_ during photoelectrochemical water oxidation^32^.

As we observed in *operando* experiment (Fig. 5a and b), a broad peak at 400–700 nm for Fe oxide under OER was related to Fe(IV) formation by some research groups^33,34^.

All the above-mentioned experiments showed that the ultra-small and highly dispersive iron oxide hydroxide was an efficient catalyst for many oxidation reactions.

Such ultra-small and highly dispersive iron oxide could be investigated to be a bridge between homogeneous and heterogeneous catalysis^12^. Among different nanomaterials, ultra-small particles (< 10 nm) show even different properties and activities than bigger particles (10–100 nm)^12,33^.

Importantly, such small iron oxides from impurity or formed by the decomposition of iron complexes can catalyze many oxidation reactions. On the other hand, such species should be carefully checked in the presence of many metal complexes since even for many pure metal complexes, the ligands are not usually stable under the harsh condition of reactions and the formation of such active metal oxides are possible^35–46^. Although an Mn oxide-based catalyst is used by Nature to oxidize water, nanosized Fe oxide shows promising activity toward OER^47,48^.

## Conclusions

Ultra-small iron oxide hydroxide (< 10 nm) was characterized by a number of methods. These methods showed that iron oxide was δ-FeOOH. Using this iron oxide, excellent conversions (75–100%) and selectivities of substrates (92–97%), depending on the nature of the sulfide, were obtained for the sulfide-oxidation reaction. The iron oxide was also applied to the oxidation of various alcohols. Benzylalcohols having electron-donating and -withdrawing substituents in the aromatic ring was oxidized to produce the corresponding aldehydes with excellent conversion (65–89%) and selectivity. A moderated activity for the epoxidation of styrene and cyclooctene was also found. A trace amount of the iron catalyst showed OER under acidic, neutral, and alkaline conditions. The slopes of Tafel plots for **1** at pH 2.0, 6.7, 12.0, and 14.0 using FTO were 361.5, 203.9, 114.0 and 124.2 mV∙decade^−1^, respectively.

### Methods

Reagents and solvents were purchased from commercial sources and were used without further purification. Ultra-small iron oxide hydroxide (FeOOH) (**1**) (< 10 nm) was purchased from Sigma-Aldrich Company. H_2_O_2_ (20%) was purchased from Merck Company. TEM was carried out with FEI Tecnai G^2^ F20 transmission electron microscope (TF20 200 kV). SEM and EDX were carried out with VEGA\TESCAN-XMU. The X-ray powder patterns were recorded with a Bruker D8 ADVANCE diffractometer (CuK_α_ radiation). Electrochemical experiments were performed using an EmStat^3+^ device from the PalmSens Company (Netherlands). For the electrochemical investigation of iron oxide catalytic behavior in water oxidation, a three-electrode cell was used. The cell was contained Ag|AgCl as a reference electrode, Pt as a counter electrode, and fluorine-doped tin oxide (Sigma-Aldrich Company, FTO) as a working electrode. The electrochemical determination was performed in bufferic phosphate solution in three different pHs (2.0, 6.7, 12.0 and 14.0). KOH was added to a solution of phosphoric acid (0.25 M) and adjusted pH in 2, 6.7, and 12.0. 5.0 µL of iron oxide mixture (20% by weight) was spread on the 1.0 cm^2^ of FTO surface. The mixture on the electrode dried at 60˚C and then 10 µL of Nafion was used to fix the solids on FTO. This electrode was placed in the cell and cyclic voltammetry at different pHs was performed. For comparison, oxygen-evolution reaction (OER) at the same pHs and surface of FTO electrode without iron oxide was determined. Thermodynamic potentials for OER in various pHs were calculated by Eqs. (2) and (3). Overpotential was calculated by Eq. (3).

2 $${\text{E}}_{{{\text{eq}}}} = 1.23{-}0.0592\;{\text{pH}}$$

3 $$\upeta = {\text{E}}_{{{\text{app}}}} {-}{\text{E}}_{{{\text{eq}}}}$$

η: Overpotential, E_appl_: Applied potential.

### General procedure for the oxidation reaction

For all oxidation experiments, we used a standard procedure. To a solution of a substrate (0.2 mmol), and **1** in water (Sigma-Aldrich Company, 67.4 μM; 1 mL), H_2_O_2_ (Merck Company, 0.4 mmol) was added as an oxidant. After four hours, water (5 mL) was added, and the resulting mixture was extracted with EtOAc (2 × 5 mL). The collected organic phases were dried with anhydrous CaCl_2_ and the extract was also concentrated down to 1.0 mL by distillation in a rotary evaporator at room temperature. Then, a sample (2 μL) was taken from the solution and was monitored by GC. Assignments of the products were made by comparison with authentic samples.

## Supplementary Information

Supplementary Information.
