# Supplementary material for: Ultra-small and highly dispersive iron oxide hydroxide as an efficient catalyst for oxidation reactions: a Swiss-army-knife catalyst
Source: Sci Rep. 2021 Mar 23;11:6642. doi: 10.1038/s41598-021-85672-x (PMC7988159; doi:10.1038/s41598-021-85672-x)
Supplement: Supplementary file 1 — Supplementary Information. [file 41598_2021_85672_MOESM1_ESM.doc]

**Supplementary Information**

**Ultra-small and highly dispersive iron oxide hydroxide as an efficient catalyst for oxidation reactions: A Swiss‐army‐knife catalyst**

Mojtaba Amini,*^a,b^ Younes Mousazade,^c^ Zahra Zand,^c^ Mojtaba Bagherzadeh,^d^ and Mohammad Mahdi Najafpour*^c,e,f^

^a^Department of Chemistry, Faculty of Science, University of Maragheh, Golshahr, P.O. Box. 55181-83111731, Maragheh, Iran

^b^Department of Inorganic Chemistry, Faculty of Chemistry, University of Tabriz, Tabriz, Iran

^c^Department of Chemistry, Institute for Advanced Studies in Basic Sciences (IASBS), 45137-66731, Zanjan, Iran

^d^Chemistry Department, Sharif University of Technology, P.O. Box 11155-3615, Tehran, Iran

^e^Center of Climate Change and Global Warming, Institute for Advanced Studies in Basic Sciences (IASBS), 45137-66731, Zanjan, Iran

^f^Research Center for Basic Sciences & Modern Technologies (RBST), Institute for Advanced Studies in Basic Sciences (IASBS), 45137-66731, Zanjan, Iran

*Corresponding authors:

mamini@maragheh.ac.ir (M.A.), Phone: (+98) 41 37278900; mmnajafpour@iasbs.ac.ir (M.M.N.), Phone: (+98) 24 3315 3201

Fig. S1 FTIR spectra of **1**.


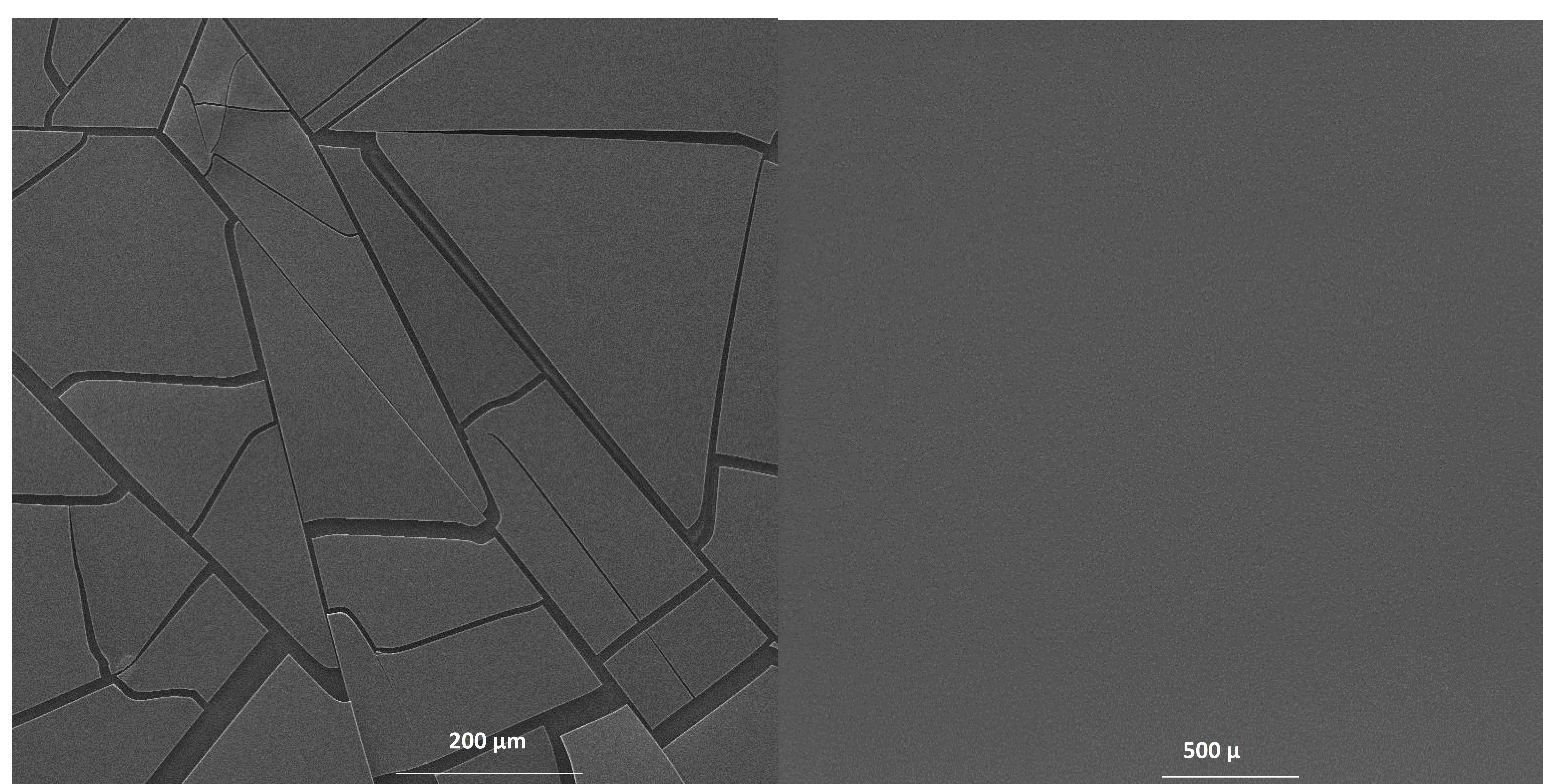


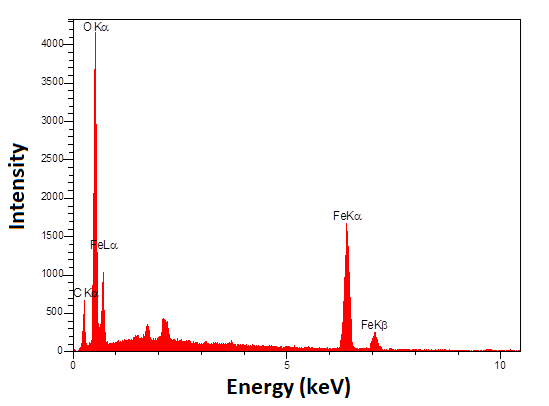


Fig. S2 SEM images at different magnifications and EDX spectrum of **1**.


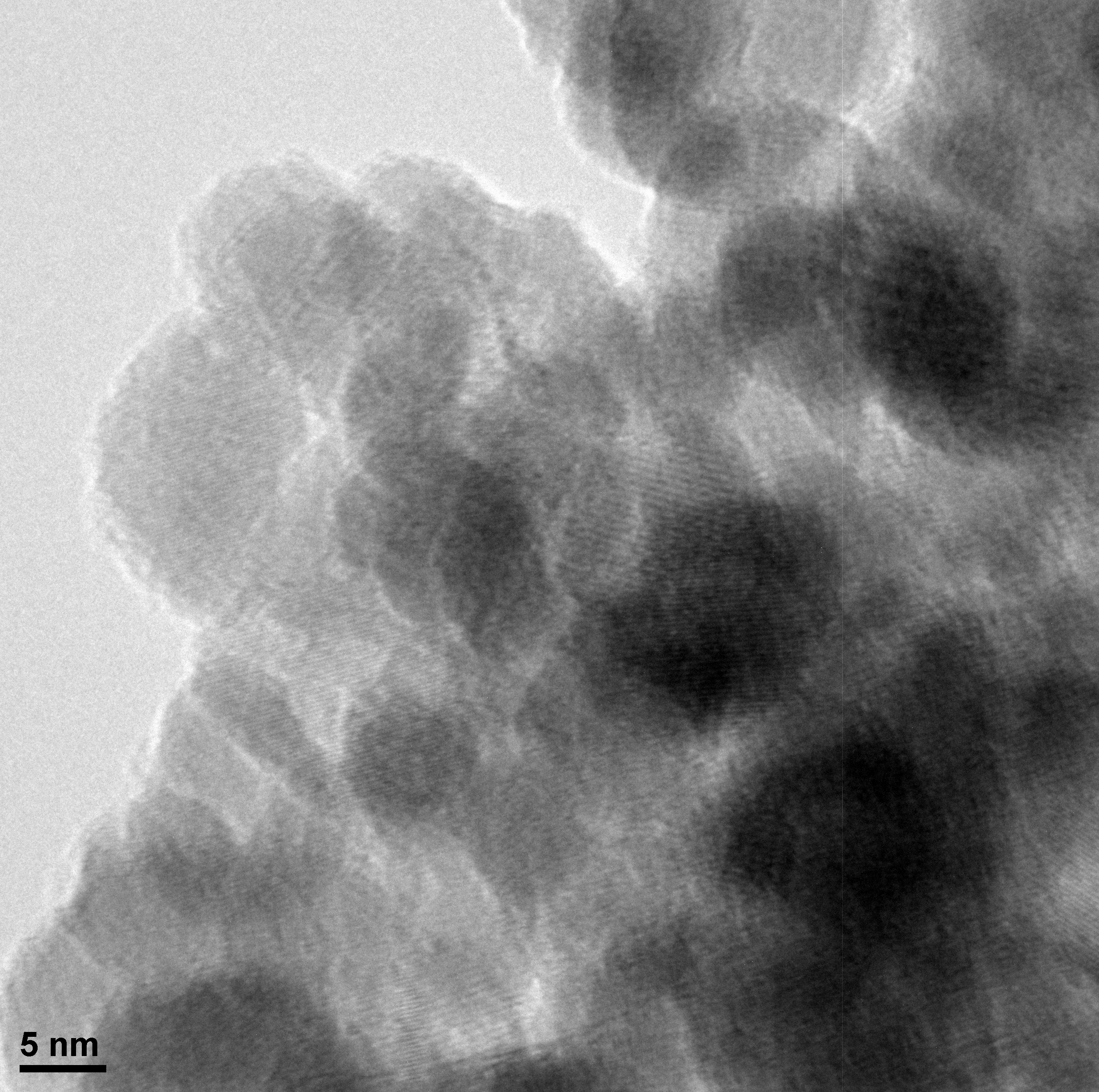

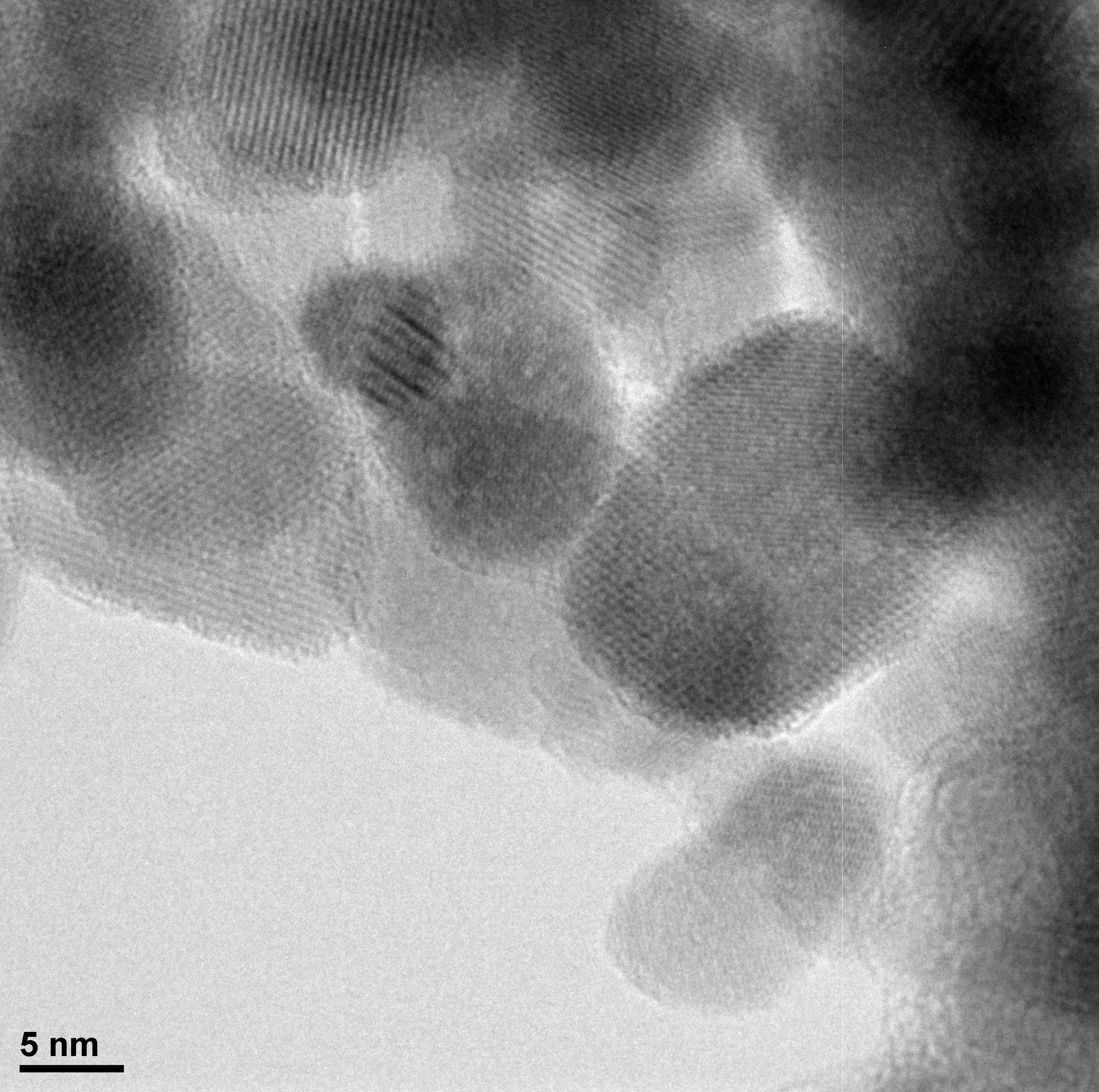

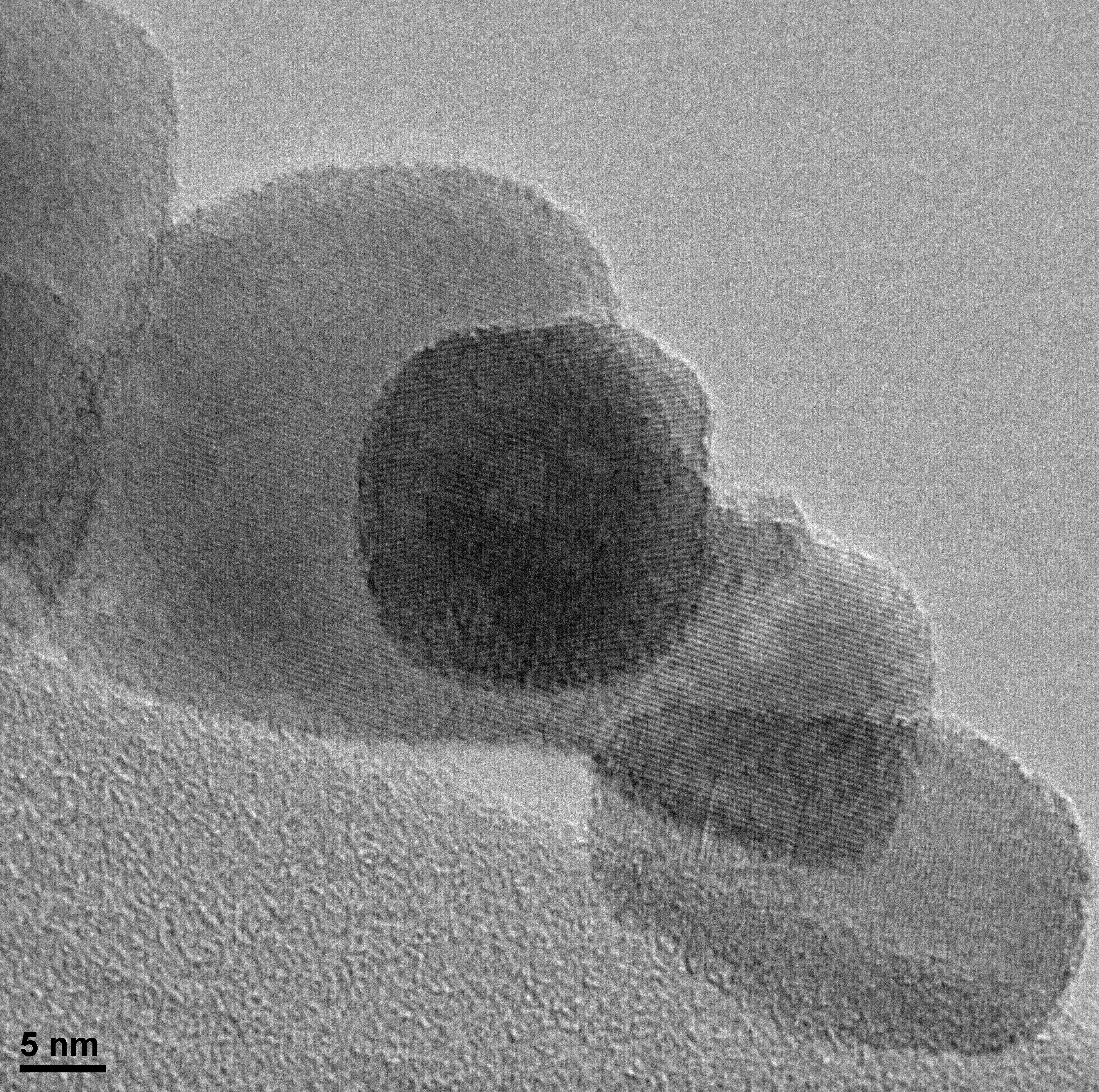

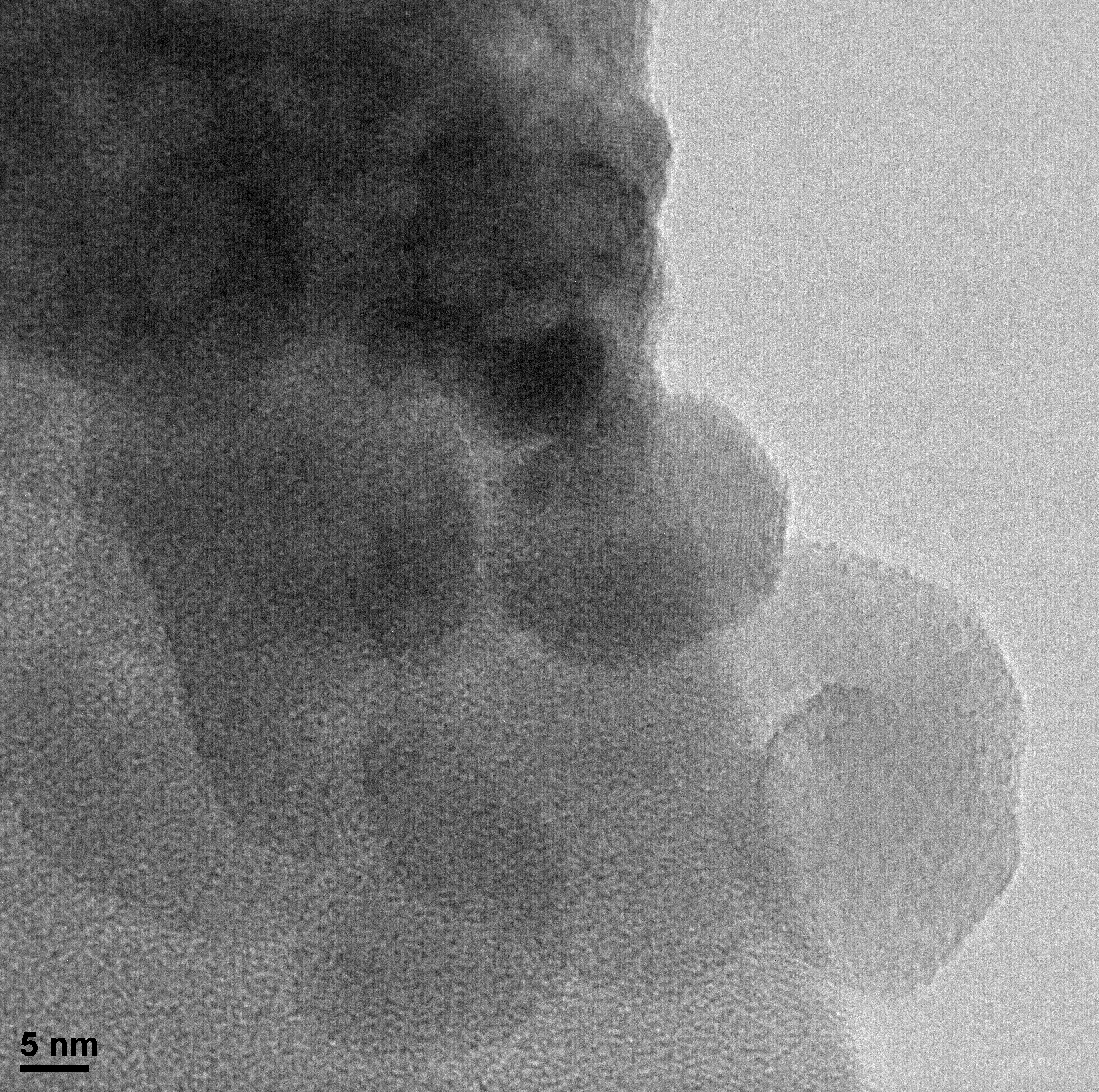


a

b

c

d

Fig. S3 TEM images of **1** at different magnifications before (a and b) and after methylphenyl sulfide oxidation (c and d).


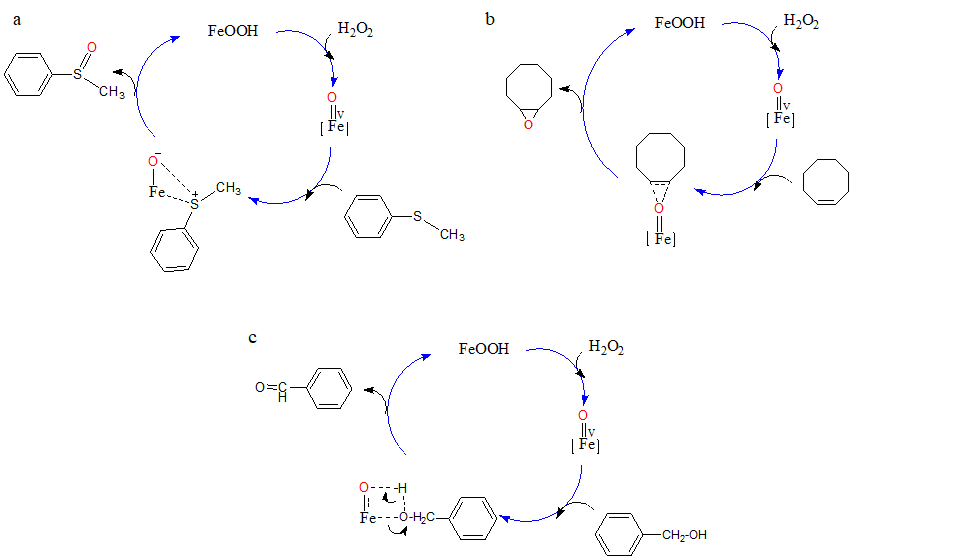


Fig. S4 Mechanism of the organic-substrates oxidation in the presence of **1**.


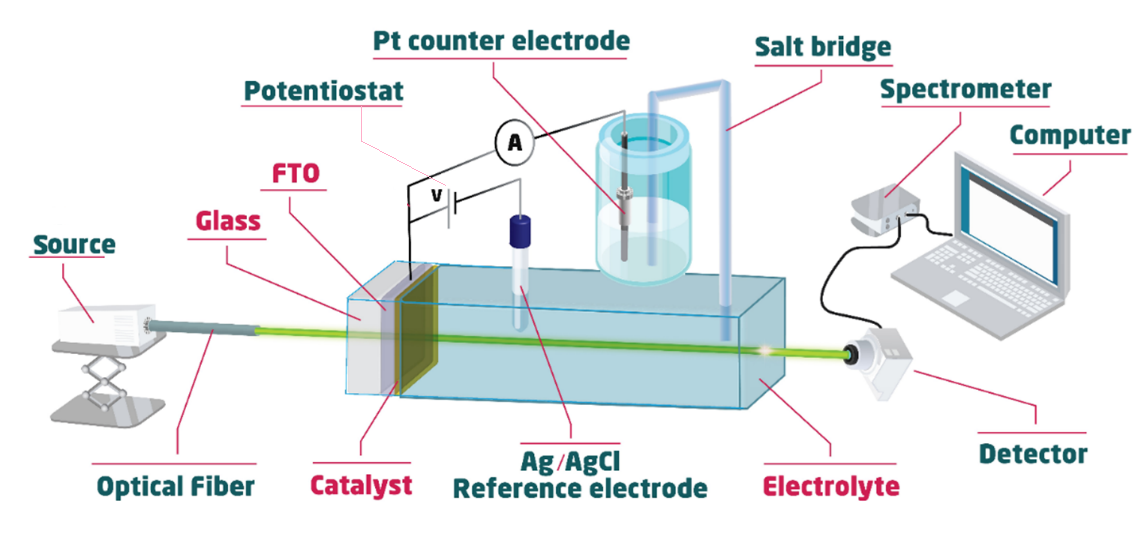


Fig. S5 Setup for high-resolution for *operando* spectroelectrochemistry.

Table S1 Comparison of some heterogeneous water-oxidizing catalysts.

| **Compound** | **η^[a]^ (mV)** | **η^[b]^ (mV)** | **pH** | **Ref.^[c]^** |
| --- | --- | --- | --- | --- |

| **This work** | **14** | **-** | **470** | **Fe(O)OH** |
| --- | --- | --- | --- | --- |
| **This work** | **12** | **830** | **640** | **Fe(O)OH** |
| **This work** | **6.7** | **910** | **690** | **Fe(O)OH** |
| **This work** | **2** | **970** | **660** | **Fe(O)OH** |
| [**^2^**](#_ENREF_2) | 14 | 297 | - | NiFeO_x_ |
| [**^3^**](#_ENREF_3) | 14 | > 1000 | > 400 | NiO_x_ |
| [**^2^**](#_ENREF_2) | 14 | 300 | - | NiO_x_ |
| [**^2^**](#_ENREF_2) | 14 | 381 | - | CoO_x_ |
| [**^2^**](#_ENREF_2) | 14 | 312 | - | NiCoO_x_ |
| [**^4^**](#_ENREF_4) | 14 | 445 | 345 | FeO_x_ |
| [**^2^**](#_ENREF_2) | 14 | 405 | - | FeO_x_ |
| [**^5^**](#_ENREF_5) | 14 | 430 | < 350 | Fe_2_O_3_ |
| [**^2^**](#_ENREF_2) | 14 | 514 | 320 | MnO_x_ |
| [**^6^**](#_ENREF_6) | 13 | - | 270 | Fe_3_Ni_2_O_x_ |
| [**^7^**](#_ENREF_7) | 13 | - | 211 | FeNiO_x_ |
| [**^8^**](#_ENREF_8) | 13 | 250 | 190 | Fe_2_Ni_3_O_x_ |
| [**^7^**](#_ENREF_7) | 13 | 280 | 191 | NiO_x_ |
| [**^9^**](#_ENREF_9) | 13 | - | 295 | NiO_x_ |
| [**^10^**](#_ENREF_10) | 13 | - | 397 | CoFeO_x_^[c]^ |
| [**^11^**](#_ENREF_11) | 13 | < 250 | < 200 | CoO_x_ |
| [**^7^**](#_ENREF_7) | 13 | 410 | 320 | FeO_x_ |
| [**^7^**](#_ENREF_7) | 13 | 270 | 210 | CoO_x_ |
| [**^6^**](#_ENREF_6) | 13 | - | 295 | CoO_x_ |
| [**^7^**](#_ENREF_7) | 13 | - | 181 | FeCoO_x_ |
| [**^7^**](#_ENREF_7) | 13 | - | 191 | FeCoNiO_x_ |
| [**^6^**](#_ENREF_6) | 13 | - | 270 | Ni_2_FeAlO_x_ |
| [**^6^**](#_ENREF_6) | 13 | - | 250 | NiFeMo_3_O_x_ |
| [**^6^**](#_ENREF_6) | 13 | - | 240 | Ni_2_FeCr_2_O_x_ |
| [**^6^**](#_ENREF_6) | 13 | - | 240 | NiFeGa_3_O_x_ |
| [**^12^**](#_ENREF_12) | 13 | 380 | 373 | CoSe_2_ |
| [**^12^**](#_ENREF_12) | 13 | 320 | 294 | NG-CoSe_2_ |
| [**^13^**](#_ENREF_13) | >11.5 | > 1000 | < 300 | MnO_x_ |
| [**^14^**](#_ENREF_14) | 11 | 420 | 300 | FeOOH |
| [**^15^**](#_ENREF_15) | 9.2 | 425 | 300 | NiB_i_ |
| [**^13^**](#_ENREF_13) | 8.5-5.5 | > 1000 | < 300 | MnO_x_ |
| [**^11^**](#_ENREF_11) | 7 | < 300 | < 200 | CoO_x_ |
| [**^16^**](#_ENREF_16) | 7 | 590 | 390 | MnO_x_ |
| [**^17^**](#_ENREF_17) | 7 | 600 | 441 | MnO_x_ |
| [**^18^**](#_ENREF_18) | 7 | > 600 | 291 | CoFePBA |
| [**^19^**](#_ENREF_19) | 7 | > 1000 | 150 | MnO_x_ |
| [**^20^**](#_ENREF_20) | 7 | 410 | 281 | CoP_i_ |
| [**^21^**](#_ENREF_21) | 7 | > 1000 | > 700 | MnO_x_ |
| [**^22^**](#_ENREF_22) | 7 | - | 500 | Li_x_MnP_2_O_7_ |
| [**^13^**](#_ENREF_13) | 3.5 | > 1000 | < 300 | MnO_x_ |
| [**^11^**](#_ENREF_11) | 1 | 600 | < 580 | Co^2+^ (1 M) |

[a] overpotential for the onset of water oxidation.

[b] @1 m/Acm^2^.

[c] For an excellent review see ref. 23

**References**

1. Xiao X.; Bard A. J. Observing single nanoparticle collisions at an ultra-microelectrode by electrocatalytic amplification. *J. Am. Chem. Soc.*, **2007**, 129, 9610-9612.

2. Trotochaud, L.; Ranney, J. K.; Williams, K. N.; Boettcher, S. W., Solution-cast metal oxide thin film electrocatalysts for oxygen evolution. *J. Am. Chem. Soc.,* **2012**, *134* (41), 17253-17261.

3. Trotochaud, L.; Young, S. L.; Ranney, J. K.; Boettcher, S. W., Nickel-iron oxyhydroxide oxygen-evolution electrocatalysts: the role of intentional and incidental iron incorporation. *J. Am. Chem. Soc.,* **2014,** *136* (18), 6744-6753.

4. Doyle, R.; Lyons, M., Kinetics and mechanistic aspects of the oxygen evolution reaction at hydrous iron oxide films in base. *J. Electrochem. Soc.,* **2013**, *160* (2), H142-H154.

5. Qiu, Y.; Leung, S.-F.; Zhang, Q.; Hua, B.; Lin, Q.; Wei, Z.; Tsui, K.-H.; Zhang, Y.; Yang, S.; Fan, Z., Efficient photoelectrochemical water splitting with ultrathin films of hematite on three-dimensional nanophotonic structures. *Nano lett.,* **2014**, *14* (4), 2123-2129.

6. Chen, J. Y.; Miller, J. T.; Gerken, J. B.; Stahl, S. S., Inverse spinel NiFeAlO_4_ as a highly active oxygen evolution electrocatalyst: promotion of activity by a redox-inert metal ion. *Energy Environ. Sci.,* **2014,** *7* (4), 1382-1386.

7. Smith, R. D.; Prévot, M. S.; Fagan, R. D.; Zhang, Z.; Sedach, P. A.; Siu, M. K. J.; Trudel, S.; Berlinguette, C. P., Photochemical route for accessing amorphous metal oxide materials for water oxidation catalysis. *Science,* **2013**, 1233638.

8. Smith, R. D.; Prévot, M. S.; Fagan, R. D.; Trudel, S.; Berlinguette, C. P., Water oxidation catalysis: electrocatalytic response to metal stoichiometry in amorphous metal oxide films containing iron, cobalt, and nickel. *J. Am. Chem. Soc.,* **2013**, *135* (31), 11580-11586.

9. Shaidarova, L.; Davletshina, L.; Budnikov, G., Flow-injection determination of water-soluble vitamins B 1, B 2, and B 6 from the electrocatalytic response of a graphite electrode modified with a ruthenium (III) hexacyanoruthenate (II) film. *J. Anal. Chem.,* **2006**, *61* (5), 502-509.

10. Abellán, G.; Carrasco, J. A.; Coronado, E.; Romero, J.; Varela, M., Alkoxide-intercalated CoFe-layered double hydroxides as precursors of colloidal nanosheet suspensions: structural, magnetic and electrochemical properties. *J. Mater. Chem. C,* **2014**, *2* (19), 3723-3731.

11. Gerken, J. B.; McAlpin, J. G.; Chen, J. Y.; Rigsby, M. L.; Casey, W. H.; Britt, R. D.; Stahl, S. S., Electrochemical water oxidation with cobalt-based electrocatalysts from pH 0–14: the thermodynamic basis for catalyst structure, stability, and activity. *J. Am. Chem. Soc.,* **2011**, *133* (36), 14431-14442.

12. Gao, M.-R.; Cao, X.; Gao, Q.; Xu, Y.-F.; Zheng, Y.-R.; Jiang, J.; Yu, S.-H., Nitrogen-doped graphene supported CoSe_2_ nanobelt composite catalyst for efficient water oxidation. *ACS Nano* **2014**, *8* (4), 3970-3978.

13. Huynh, M.; Bediako, D. K.; Nocera, D. G., A functionally stable manganese oxide oxygen evolution catalyst in acid. *J. Am. Chem. Soc.,* **2014**, *136* (16), 6002-6010.

14. Chemelewski, W. D.; Lee, H.-C.; Lin, J.-F.; Bard, A. J.; Mullins, C. B., Amorphous FeOOH oxygen evolution reaction catalyst for photoelectrochemical water splitting. *J. Am. Chem. Soc.,* **2014**, *136* (7), 2843-2850.

15. Dincă, M.; Surendranath, Y.; Nocera, D. G., Nickel-borate oxygen-evolving catalyst that functions under benign conditions. *Proc. Natl. Acad. Sci.,* **2010**, *107* (23), 10337-10341.

16. Zaharieva, I.; Chernev, P.; Risch, M.; Klingan, K.; Kohlhoff, M.; Fischer, A.; Dau, H., Electrosynthesis, functional, and structural characterization of a water-oxidizing manganese oxide. *Energy Environ. Sci.,* **2012**, *5* (5), 7081-7089.

17. Indra, A.; Menezes, P. W.; Zaharieva, I.; Baktash, E.; Pfrommer, J.; Schwarze, M.; Dau, H.; Driess, M., Active Mixed‐Valent MnO_x_ Water Oxidation Catalysts through Partial Oxidation (Corrosion) of Nanostructured MnO Particles. *Angew. Chem. Int. Ed.,* **2013**, *52* (50), 13206-13210.

18. Pintado, S.; Goberna-Ferrón, S.; Escudero-Adán, E. C.; Galán-Mascarós, J. R. n., Fast and persistent electrocatalytic water oxidation by Co-Fe Prussian blue coordination polymers. *J. Am. Chem. Soc.,* **2013**, *135* (36), 13270-13273.

19. Singh, A.; Hocking, R. K.; Chang, S. L.-Y.; George, B. M.; Fehr, M.; Lips, K.; Schnegg, A.; Spiccia, L., Water oxidation catalysis by nanoparticulate manganese oxide thin films: probing the effect of the manganese precursors. *Chem. Mater.,* **2013**, *25* (7), 1098-1108.

20. Kanan, M. W.; Nocera, D. G., In situ formation of an oxygen-evolving catalyst in neutral water containing phosphate and Co^2+^. *Science*, **2008**, *321* (5892), 1072-1075.

21. Bergmann, A.; Zaharieva, I.; Dau, H.; Strasser, P., Electrochemical water splitting by layered and 3D cross-linked manganese oxides: correlating structural motifs and catalytic activity. *Energy Environ. Sci.*, **2013**, *6* (9), 2745-2755.

22. Park, J.; Kim, H.; Jin, K.; Lee, B. J.; Park, Y.-S.; Kim, H.; Park, I.; Yang, K. D.; Jeong, H.-Y.; Kim, J., A new water oxidation catalyst: lithium manganese pyrophosphate with tunable Mn valency. *J. Am. Chem. Soc.,* **2014**, *136* (11), 4201-4211.

23 Galan-Mascarós, J. R., Water oxidation at electrodes modified with earth‐abundant transition‐metal catalysts. *ChemElectroChem*, **2015**, 2 (1), 37-50.
